# Transcriptional Regulation of the Beta-Synuclein 5′-Promoter Metal Response Element by Metal Transcription Factor-1

**DOI:** 10.1371/journal.pone.0017354

**Published:** 2011-02-28

**Authors:** Patrick C. McHugh, Josephine A. Wright, David R. Brown

**Affiliations:** Department of Biology and Biochemistry, University of Bath, Claverton Down, Bath, United Kingdom; University Medical Center Groningen, University of Groningen, Netherlands

## Abstract

The progression of many human neurodegenerative disorders is associated with an accumulation of alpha-synuclein. Alpha-synuclein belongs to the homologous synuclein family, which includes beta-synuclein. It has been proposed that beta-synuclein may be a natural regulator of alpha-synuclein. Therefore controlling beta-synuclein expression may control the accumulation of alpha-synuclein and ultimately prevent disease progression. The regulation of synucleins is poorly understood. We investigated the transcriptional regulation of beta-synuclein, with the aim of identifying molecules that differentially control beta-synuclein expression levels. To investigate transcriptional regulation of beta-synuclein, we used reporter gene assays and bioinformatics. We identified a region −1.1/−0.6 kb upstream of the beta-synuclein translational start site to be a key regulatory region of beta-synuclein 5′-promoter activity in human dopaminergic cells (SH-SY5Y). Within this key promoter region we identified a metal response element pertaining to a putative Metal Transcription Factor-1 (MTF-1) binding site. We demonstrated that MTF-1 binds to this 5′-promoter region using EMSA analysis. Moreover, we showed that MTF-1 differentially regulates beta-synuclein promoter binding site, as well as beta-synuclein mRNA and protein expression. This effect of MTF-1 on expression was found to be specific to beta-synuclein when compared to alpha-synuclein. Understanding the regulation of synucleins and how they interact may point to molecular targets that could be manipulated for therapeutic benefit. In this study we showed that MTF-1 differentially controls the expression of beta-synuclein when compared to its homolog alpha-synuclein. This could potentially provide a novel targets or pathways for therapeutic intervention and/or treatment of synucleinopathies.

## Introduction

Neurodegenerative disorders constitute a disease category that the World Health Organization calculates will become the world's second leading cause of death by the year 2040, overtaking cancer. The progression of many human neurodegenerative disorders is associated with an accumulation of alpha-synuclein (α-Syn), including Alzheimer's disease (AD) and Parkinson's diseases (PD), dementia associated with Lewy body disease (DLB), diffuse Lewy body disease (DLBD) and multiple system atrophy (MSA). This group of diseases are now known as synucleinopathies and are characterised by the presence of Lewy bodies (LB), the intracytoplasmic inclusions/aggregates found in dopaminergic neurons containing alpha-synuclein (α-Syn) [1]. Abnormal protein-protein interactions may allow the precipitation of α-Syn, which facilitates the formation of these extracellular and intracellular aggregates. The formation of these deposits can be induced by a number of substances, including metal ions [2]. Indeed, high levels of metals have been identified in the substantia nigra of PD brains [3]. Aggregated α-Syn potentially inhibits proteasomal activity [4], which may be a cause of the observed reduction in proteasome activity in the substantia nigra of PD patients [5]. Studies in families affected by dementia, PD and DLBD have genomic amplifications encompassing the α-Syn gene, leading to a proportional increase in α-Syn mRNA and protein in brain tissue [6]–[8]. These cases support the hypothesis that increased expression of α-Syn causes disease.

α-Syn belongs to the homologous synuclein family, which includes beta-synuclein (β-Syn). Expression patterns and levels of α-Syn and β-Syn most closely overlap with highest levels throughout the brain [9]. Although β-Syn is not detected in LB or form fibrils like α-Syn [10], β-Syn mutants have been identified in DLB patients [11] and β-syn protein is found to be abundant in neurofibrillary lesions of patients with AD [12]. Moreover, in both the mouse brain and the human substantia nigra, α-Syn mRNA decreases and β-Syn mRNA increases with age [13]. In contrast to control patients, there is a dramatic increase in α-Syn and decrease in β-Syn mRNA levels in the substantia nigra of PD, DLBD and a LB variant of AD patients [14]. These changes were specific for the substantia nigra, the dopaminergic neuron-containing region of the brain most severely affected in synucleinopathies. This concentration reversal of synuclein transcript levels with disease suggests the balance of α-Syn and β-Syn expression may be important, which is supported by several studies [15], [16]. This suggests that β-Syn may be a natural negative regulator of α-Syn.

Transcriptional Regulation of the synucleins has been reported. Polymorphisms of the dinucleotide repeat complex NACP-Rep1 (10.7 kb upstream of the translational start site are associated with AD and PD [17], [18]. Different NACP-REP1 alleles have varying repressive effects on α-Syn promoter driven reporter activity in SH-SY5Y cells [19]. Assessment of regions of the α-Syn promoter suggest the presence of activator sites between −1.5/−1.9 kb and repressor sites between −6.2/−9.8 kb upstream of the translational start site [19]. Regulators of β-Syn expression have not been reported. Since β-Syn may be a natural negative regulator of α-Syn, identifying transcriptional regulators of β-Syn is essential. In particular, β-Syn is a good target for disease therapies as there are no apparent abnormalities when it is overexpressed in transgenic mice [16]. Manipulation of transcriptional regulators of β-Syn expression would increase β-Syn levels, inhibit aggregation of α-Syn and potentially prevent disease progression. This study aimed to investigate the regulation of the β-Syn promoter.

## Materials and Methods

### Tissue Culture

Two cell lines were used in our promoter analysis, SH-SY5Y neuroblastoma cells (ATCC (VA, USA) # CRL-2266) and U-87 MG astrocytoma cells (ECACC (Sigma-Aldrich Company Ltd, Poole, UK) # 89081402). All tissue culture reagents and plastic-ware were obtained respectively from Lonza Biologics plc (Slough, UK) and Greiner Bio-One Ltd (Stroudwater, UK) unless otherwise specified. SH-SY5Y cells were maintained in a 50/50 mix of DMEM (#BE12-604F) and HAMS F12 (#BE12-615F) with 10% fetal calf serum in 75 cm^2^ flasks at 37°C (5% CO_2_). U-87 MG cells were maintained in EMEM (ATCC #30-2003) with 10% foetal calf serum in 75 cm^2^ flasks at 37°C (5% CO_2_).

### PCR and Cloning

Several β-Syn promoter constructs of varying length and the open reading frames of α-syn, β-Syn, γ-Syn and MTF-1 were cloned. Briefly, PCR products were generated using primers into which restriction endonuclease sites were engineered (Table 1). For the promoter constructs, PCRs were performed using a genomic clone that contained the beta-synuclein gene (ImaGenes, RZPDB737). For mammalian protein expression analysis, PCRs were performed using cDNA generated from SH-SY5Y cells, these methods have been previously described [20].

**Table 1:** Oligonucleotides used for cloning.

Construct a	Sequence (5′ to 3′) b	RE
−10.8/−8.3	F - ATAT**CTCGAG**CCACCCCAAACTCAGTGG	*Xho*I
	R - ATTT**GGATCC**GGGAGAACTGGTCATGGC	*Bam*HI
−10.8/−6	F - ATAT**CTCGAG**CCACCCCAAACTCAGTGG	*Xho*I
	R - TAAA**GGTACC**CAAGATGGCTGCTGCAGC	*Kpn*I
−6/−0.9	F - ATAT**CTCGAG**GCTGCAGCAGCCATCTTG	*Xho*I
	R - ATTT**GGATCC**CTTCGGCCTGACTGACAG	*Bam*HI
−6/−3.7	F - ATAT**CTCGAG**GCTGCAGCAGCCATCTTG	*Xho*I
	R - TAAA**GGATCC**GAGACTACAGGTGCGTGC	*Bam*HI
−3.7/ATG	F - ATAT**CTCGAG**GCACGCACCTGTAGTCTC	*Xho*I
	R - TAAA**GGATCC**GAACACGTCCATCCTGGC	*Bam*HI
−3.7/−0.9	F - ATAT**CTCGAG**GCACGCACCTGTAGTCTC	*Xho*I
	R - ATTT**GGATCC**CTTCGGCCTGACTGACAG	*Bam*HI
−1.1/−0.6	F - ATAT**CTCGAG**CAGCTGAGATTCGAGCCC	*Xho*I
	R - ATTT**GGATCC**GCCGCCTCACCTGGATGC	*Bam*HI
−0.9/ATG	F - ATAT**CTCGAG**CTGTCAGTCAGGCCGAAG	*Xho*I
	R - TAAA**GGATCC**GAACACGTCCATCCTGGC	*Bam*HI
−0.6/ATG	F - ATAT**CTCGAG**CTGTCAGTCAGGCCGAAG	*Xho*I
	R - TAAA**GGATCC**GAACACGTCCATCCTGGC	*Bam*HI
β-Syn	F - GACGAGT**AAGCTT**GACGTGTTCATGAAGGG	*Hind*III
	R - AAATAT**GGATCC**TTACTACGCCTCTGGCTCATAC	*Bam*HI
γ-Syn	F - GACGAGT**AAGCTT**GATGTCTTCAAGAAGGG	*Hind*III
	R - AAATAT**GGATCC**TTACTAGTCTCCCCCACTCTG	*Bam*HI

**a** pEGFP-PrP (prion protein) was a gift from Dr Catia Sorgato and was subcloned into pcDNA3.1+ using *Hind*III and *Eco*R1 restriction sites and is not listed. pcDNA3.1+α-Syn, pcDNA 3.1+*NRF2*, pcDNA3.1+*MTF-1* have been described previously [50], [51] and are not listed.

**b** Restriction endonuclease recognition sites are bolded and underlined; each oligonucleotide has a 4–7 bp flanking tag.

RE  =  Restriction Endonuclease.

PCRs were carried out in a total volume of 50 µl, containing 200 µmol/L dNTPs, 0.4 µM of each primer, 1.5 mmol/L MgCl_2_, 1U of Phire *Taq* DNA Polymerase (Finnzymes, Labtech International LTD, UK), 1 µl reaction buffer, and 20 to 100 ng of DNA. Thermal cycling conditions were as follows: 2 min at 94°C (initial denaturation); 35 cycles of 30 s at 94°C, 30 s at 55–65°C, 40 s at 72°C; and a final extension of 2 min at 72°C. PCR products were cloned into either the luciferase promoter reporter vector, pGL3-basic (Promega Corporation, UK) or the mammalian expression vector, pcDNA3.1+ (Invitrogen, UK). Constructs were confirmed by restriction endonuclease digestions and DNA sequencing and large scale DNA preparations were made with GeneJET™ Pasmid Miniprep Kit (Fermentas, UK).

### Promoter Assays

Transfections into SH-SY5Y and U-87 MG cells were performed in 24-well plates seeded at 5×10^4^ cells/well 24 h prior to transfection. Transfections were performed using FuGENE6® transfection reagent (Roche, Mannheim, Germany) as per manufacturer's instructions. To control for variation in transfection efficiency among replicates, promoter constructs were co-transfected with the *Renilla* luciferase vector, pRL-TK, (Promega). At 48 h post-transfection, SH-SY5Y and U-87 MG cells were harvested and firefly and *Renilla* luciferase chemiluminescence were measured using the Dual-Luciferase® Reporter Assay System (Promega) in a BMG FLUOstar Omega platereader (BMG Labtech GmbH, Offenburg, Germany). Luciferase activity was calculated as the ratio of firefly to *Renilla* luciferase activity. Each construct was tested in triplicate with at least three independent transfection experiments.

For experiments investigating copper, SH-SY5Y cells were plated 24 hr prior to transfection in the presence of serum-free media. To reduce copper levels we replaced fetal calf serum with B27 supplement (Invitrogen). The concentration of copper in defined medium is 0.1 µM [21]. Cells were then supplemented with no copper, 10 µM or 50 µM at time of transfection.

### Site-Directed Mutagenesis

Site-directed mutagenesis was used to introduce a point mutation at −774 bp of the putative β-Syn promoter, specifically within a metal response element (MRE). PWO polymerase (Roche) was used with the following oligonucleotides in PCR reactions as per manufacturers instructions: Forward, 5′-CCGGGCACTGCTGCGTTCCTGCTCAG-3′; Reverse, 5′-GTCCTGAGCAGGAACGCAGCAGTGC-3′. MRE is shown in bold with mutant base underlined. Thermal cycling conditions were as follows: 2 min at 94°C (initial denaturation); 35 cycles of 30 s at 94°C, 30 s at 55–65°C, 40 s at 72°C; and a final extension of 2 min at 72°C.

### Electrophoretic Mobility Shift Assays (EMSAs)

MTF-1 transcription factor binding to the MRE was determined by using EMSA. SH-SY5Y cells were stably transfected with empty expression vector (pcDNA3+) or pcDNA3.1+MTF-1. Cells were transfected as described above, except after 24 h transfection, cells containing vectors were selected for using media containing 400 µg/ml geneticin (Sigma). After several rounds of selection and maintenance, cells were washed in ice cold PBS and the protein was extracted using the NE-PER Nuclear and Cytoplasmic Extraction kit (Thermo Scientific, UK). Protein was quantified using the Bradford assay (Bio-Rad Laboratories Ltd, Hercules, CA). Synthetic biotin and non-biotin labelled probes (MWG, Germany) for the MRE (shown in bold) for normal MRE (Forward, 5′-CCCGGGCACTGC**TGCGCTC**CTGCTCAGGACC-3′; Reverse, GGTCCTGAGCAG**GAGCGCA**GCAGTGCCCGGG-3′) and mutant MRE (Forward, 5′- CCCGGGCACTGC**TGCGTTC**CTGCTCAGGACC-3′; Reverse, 5′- GGTCCTGAGCAG**GAACGCA**GCAGTGCCCGGG-3′) were obtained for EMSA analysis. Binding reactions containing equal amounts of protein (15 µg) and probes were performed using the LightShift® Chemiluminescent EMSA kit (Thermo Scientific, UK) as permanufacturer's instructions. Protein-DNA complexes were electrophoresed on 6% non-denaturing polyacrylamide gels, and transferred to Hybond-N+ (GE Healthcare, UK); signals were detected using LightShift® Chemiluminescent EMSA kit (Thermo Scientific). All EMSA assays were performed for three sample replicates in each group.

### Quantitative PCR

Cells stably transfected with pcDNA3.1+ or pcDNA3.1+MTF-1 were washed in ice cold PBS and RNA was obtained using TRI reagent (Sigma) as per manufacturer's instructions. Five micrograms of RNA was used from each sample for the synthesis of cDNA in a 20 µl volume with oligo(dT)_20_ (Invitrogen) and SuperScript III reverse transcriptase (Invitrogen). The reactions were incubated at 50°C for 1 hr and then inactivated by heating at 70°C for 15 min. RNaseH (Invitrogen) was added, 1 µl to each preparation, and incubated for 20 min at 37°C. Six microlitres of diluted cDNA was used as template in quantitative PCR (QPCR) reactions in a 15 µl volume with 7.5 µl iTaq SYBR Green™ Supermix with ROX (Bio-Rad), 0.3 µl 100 nM of each primer (Table 2). Primers were designed, where possible, to span exons and to generate a fragment of between 80 and 130 bp. The QPCR assays were performed on a the Step One Plus™ Real-Time PCR System (Applied Biosystems, Foster City, CA) using the following PCR conditions: 2 min at 95°C followed by 40 cycles of 15 s denaturing at 95°C and 1 min annealing at 60°C and 15 s acquiring the fluorescence data (temperature dependent on the gene being assayed). A RT-negative control was used to assess whether any residual genomic DNA remained and a no-template control was included to examine primer-dimer formation. QPCR experiments were excluded for analysis if the reaction efficiency was outside the range of 0.9 – 1.1 or if the r^2^ value of the linear regression was less than 0.95. Furthermore, the inter-experiment variability between replicates was assessed by measuring sample coefficient of variation and excluding samples if this was over 30%.

**Table 2:** Oligonucleotides used for QPCR.

Gene	Sequence (5′ to 3′)	Amplicon Size (bp)
*ACTB*	F - AGCAAGCAGGAGTATGACGAG	92
	R - AAGAAAGGGTGTAACGCAACTAA	
*GAPDH*	F - AATCCCATCACCATCTTCCA	122
	R - AAATGAGCCCCAGCCTTC	
*β2M*	F - ATTCTACTTTGAGTGCTGTCTCCA	101
	R - ATTCTCTGCTCCCCACCTCT	
*SNCA*	F - CTGCCCCCACTCAGCATT	88
	R - AAGCCACAAAATCCACAGCA	
*SNCB*	F - GGAGCCAGAAGGGGAGAGT	119
	R - GCAGGGACAGGGACAGAA	
*SNCG*	F - AAAGAGGAAGTGGCAGAGGA	99
	R - GATGGTGTCCAAGGCAGAG	

For the QPCR analysis, the relative quantification method was used. Our approach incorporated a standard curve to allow determination of the relative number of cDNA molecules of a given gene by using serial dilutions of an arbitrary cDNA sample. Expression of the gene of interest was normalised by the geometric mean of expression from three reference genes (*GAPDH, ACTB* and *B2M*). QPCR was performed with cDNA samples in triplicate from five independent replicates of cDNA from SH-SY5Y cells over-expressing various proteins.

### Western Immunoblotting

Cells stably transfected with pcDNA3.1+ or pcDNA3.1+MTF-1 were washed in ice cold PBS and the protein was obtained as described above. Thirty micrograms of cytosolic protein was used for identifying α-Syn (Roche) and β-Syn (Millipore, UK) protein levels. All experiments were performed in triplicate. Protein band intensities were measured using Quantity One densitometry software (Bio-Rad). Further details of Western immunoblot analysis have been described previously [22].

### Statistical Analysis

We evaluated statistical significance for the promoter assays, QPCR and Western immunoblot analysis with paired *t-*tests on GraphPad Prism software (GraphPad, San Diego, CA).

## Results

### Basal promoter activity

**Figure 1:**
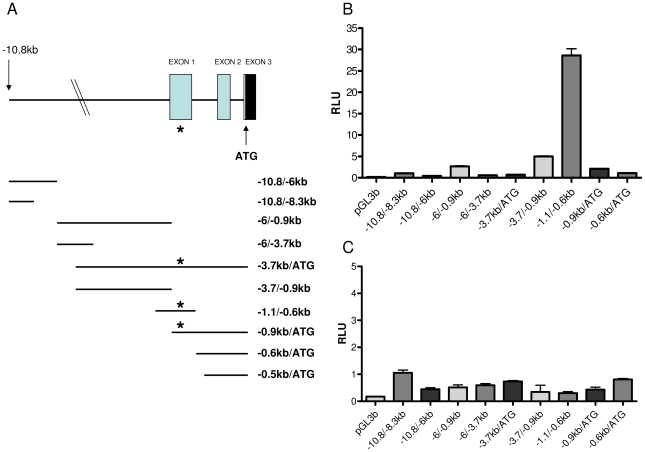
β-Synuclein 5′- genomic structure and basal promoter activity. (A) Depicts the 5′-genomic structure of human β-Synuclein gene. Exons are depicted as closed boxes, and 5′-region and introns are black lines. Black box indicates the translational start site in exon 3. Lower panel, luciferase constructs ranging across the 5′-region. * Indicates metal response element (MRE- TGCGCTC). (B & C) Reporter gene assays using Dual-Luciferase™ shows the basal activity of β-Syn promoter fragments and empty pGL3basic vector in SH-SY5Y and U87 MG cells respectively. RLU  =  relative luciferase units.

The putative β-syn promoter region was assessed using a Dual-Luciferase® reporter assay system. The genomic organisation of β-Syn has previously been described [23]. A region spanning ∼10.8 kb 5-prime of the translational start site, pertaining to nine promoter fragments, were analysed (Figure 1A). Parallel reporter gene assays of the nine constructs demonstrated a significant difference in basal transcription rate, in SH-SY5Y cells, between pGL3b −1.1/−0.6 kb and the other β-syn promoter constructs, which had minimal or negligible activity above background (Figure 1B). Background is measured by empty pGL3basic vector. All the β-Syn promoter constructs had minimal or negligible activity in the U-87 MG astrocytoma cells (Figure 1C).

### Transcription Factor regulation of the β-Syn promoter

**Figure 2:**
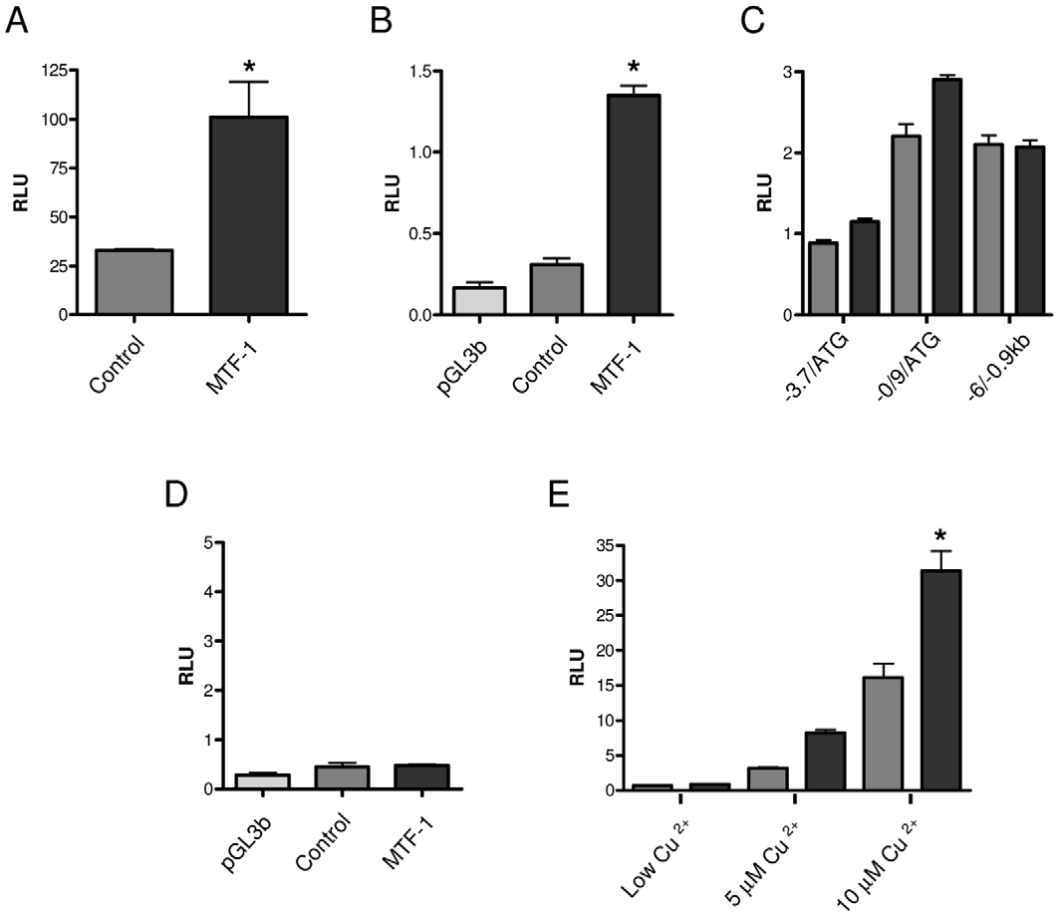
β-Syn promoter regulation. The activity of β-Syn promoter fragment pGL3b −1.1/−0.6 kb in cells stably transfected with pcDNA3.1+ or pcDNA3.1+MTF-1 in (A) SH-SY5Y and (B) U87 MG cells. (C) The activity of β-Syn promoter fragments pGL3b −3.7 kb/ATG, −0.9 kb/ATG and −6.0/−0.9 kb in SH-SY5Y cells over-expressing pcDNA3.1+ or pcDNA3.1+MTF-1. (D) The activity of β-Syn promoter fragment pGL3b −1.1/−0.6 kb with mutation inserted at −774 bp in SH-SY5Y cells over-expressing pcDNA3.1+ or pcDNA3.1+MTF-1. (E) The activity of β-Syn promoter fragment pGL3b −1.1/−0.6 kb in the presence of 0 µM, 10 µM & 50 µM copper in cells stably transfected with either pcDNA3.1+ or pcDNA3.1+MTF-1 The pGL3basic background activity is shown in graphs where the promoter activity is low. *  =  *p*<0.05, all the rest were non-significant. RLU  =  relative luciferase units. Light grey bars  =  pGL3basic; dark grey bars  =  pcDNA3.1+; black bars  =  pcDNA3.1+MTF-1.

To investigate the regulation of β-Syn −1.1/−0.6 kb, we performed a transcriptional binding site analysis using the Transcription Element Search System (TESS) [24] and identified a putative metal transcription factor-1 (MTF-1) binding site pertaining to a metal response element (MRE-TGCGCTC). To explore this further we looked at the effect of cells over-expressing MTF-1 on the β-Syn −1.1/−0.6 kb promoter fragment. Figure 2A shows that in cells over-expressing pcDNA3.1+MTF-1 there was a statistically significant increase in β-Syn −1.1/−0.6 kb promoter stably-transfected with the empty expression vector, pcDNA3.1+. We also tested the affect of MTF-1 on −1.1/−0.6 kb in U-87 MG cells (Figure 2B) and although the basal activity of −1.1/−0.6 kb was only slightly above empty vector (pGL3basic), there was a similar trend for that observed in the SH-SY5Y cells. We also investigated whether MTF-1 affected two larger promoter constructs containing the MRE (−3.7kb/ATG & -0.9 kb/ATG) and a larger fragment without the MRE (−6.0/−0.9 kb) (Figure 2C). All three constructs were not significantly affected by MTF-1 over-expression, however, the two MRE-containing promoter fragments did trend to the effect observed for the β-Syn −1.1/−0.6 kb construct. This less dramatic effect on the two larger MRE-containing promoter fragments could be due to the presence of repressor elements counteracting the effect of MTF-1 on the MRE, which may also explain the initial lower basal activity observed for these two promoter constructs (Figure 1B).

We investigated whether the addition of copper from a low basal level affected the activity of the −1.1/−0.6 kb promoter construct and found that in serum-free media, where copper levels are 5.2 nM [25], the activity of the −1.1/−0.6 kb fragment was abolished, even in the presence of over-expressed MTF-1 (Figure 2E). When we added copper at 10 µM and 50 µM the basal activity was restored, as well as MTF-1 induction of the promoter, although the overall activity was not as high as in normal conditions (Figure 2A). In normal serum media the concentration of copper is ∼2 µM, suggesting that other factors in normal serum, not present in the B27 supplement, maybe important for normal promoter activity. These results suggest that copper is important for proper β-syn promoter function, as well as MTF-1 regulation of the promoter.

### MTF-1 binding to the β-Syn promoter MRE

To assess whether this increase in promoter activity was specific to the MTF-1 binding site or an indirect effect of MTF-1 over-expression, we abolished the MRE binding site in β-Syn −1.1/−0.6 kb by site-directed mutagenesis. Figure 2D shows that the inserted mutation at −774 bp of the β-syn promoter, corresponding to the putative MTF-1 binding site, abolishes promoter activity to near basal levels, regardless of MTF-1 over-expression, as can be seen when compared to pGL3basic. Therefore, it appears that this binding site is critical for β-Syn promoter activity.

**Figure 3:**
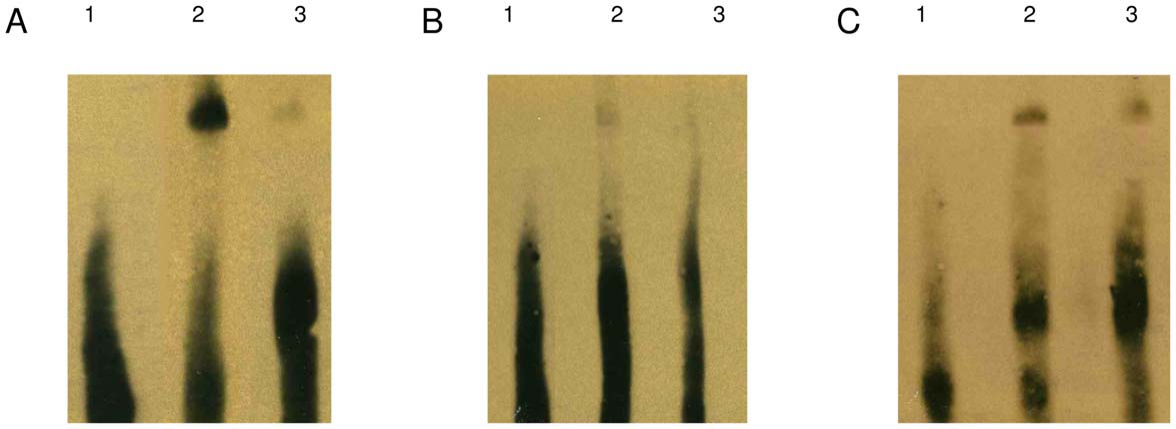
EMSA analysis. Nuclear extracts from SH-SY5Y cells were stably transfected with (A & C) pcDNA3.1+MTF-1 or (B) pcDNA3.1+. A & B are probed with normal probe and C with mutant probe. Lane 1 is the migration of free-probe in the absence of nuclear extract and therefore no shift observed. Lane 2 is either the MRE-containing double-stranded DNA probe (A & C) and shows a signal shift due to transcription factor binding or the mutant MRE-containing probe (B) and shows a diminished signal shift. Lane 3 shows that the signal shift can be inhibited from excess non-labelled probe.

To determine whether the observed MTF-1 regulation of the β-Syn promoter was a consequence of MTF-1 binding to the β-syn promoter MRE or an indirect effect of MTF-1 on the β-syn promoter activity, we performed EMSAs. EMSA analysis showed that in cells over-expressing the transcription factor MTF-1 there was an increase in binding for the normal MRE-containing probe compared to the mutant probe as indicated by the band intensity shift when compared to unlabelled probe (Figure 3A & B). In comparison to pcDNA3.1+ over-expressing cells, this effect was less intense (Figure 3C), although more intense than the mutant probe in cells over-expressing MTF-1, suggesting that endogenous levels of MTF-1 are present. These results show that MTF-1 binds to MRE located in the −1.1/−0.6 kb region of the β-Syn 5′-promoter.

### MTF-1 regulates β-Synuclein expression

**Figure 4:**
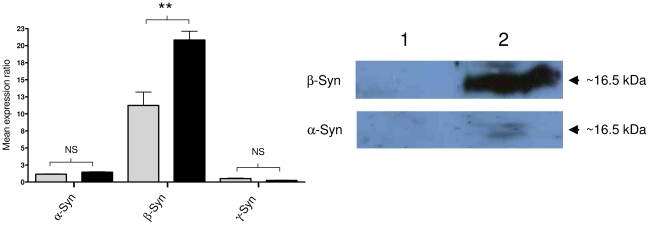
Expression analysis. (A) QPCR analysis. Normalised mean of differences in expression between control (grey bars) and MTF-1 (black bar) over-expressing SH-SY5Y cell samples for α-Syn, β-Syn and γ-Syn genes. Control  =  pcDNA3.1+ (empty expression vector). The mean expression ratios ± standard error for triplicate experiments are as follows; α-Syn - pcDNA3.1+ (1.17±0.04) & MTF-1 (1.45±0.07), β-Syn - pc (11.24±1.96) & MTF-1 (20.84±1.26) and γ-syn - pcDNA3.1+ (0.52±0.08) & MTF-1 (0.25±0.05). NS  =  non-significant; **  =  p<0.01. (B) Western immunoblotting. Cytosolic extracts from SH-SY5Y cells stably-transfected with pcDNA3.1+ (Lane 1) or MTF-1 (Lane 2) were probed with either α-Syn or β-Syn. Band intensity was measured using Quantity One densitometry software (Bio-Rad). The mean intensity values ± standard error and statistical significance for triplicate experiments are as follows; β-Syn - Lane 1 (144±23) & Lane 2 (4276±216) (p = 0.0024), α-Syn - Lane 1 (202±48) & Lane 2 (362±44) (p = 0.1714).

We looked to see whether the observed interaction of MTF-1 with the β-Syn promoter, as well as the increase in promoter activity due to over-expression of MTF-1, affected mRNA and protein levels. QPCR analysis was performed to see whether over-expression of MTF-1 affected β-Syn transcription levels. Figure 4A shows that over-expression of MTF-1 has a statistically significant effect on β-Syn gene expression when compared to control cells stably transfected with the empty vector, pcDNA3.1+. This effect of MTF-1 on gene expression is specific to β-Syn as shown when compared to the effect on the other synucleins, α-Syn and γ-Syn (Figure 4A).

Western immunoblot analysis shows that in cells over-expression of MTF-1 there was a significant effect on β-Syn protein expression, with a 30-fold increase in protein abundance, when compared to control cells (Figure 4B). For comparison, we also looked at the effect of MTF-1 over-expression on α-Syn protein levels and found no significant change in expression.

## Discussion

We analysed the beta-Synuclein (β-Syn) putative 5′-promoter and identified a region −1.1/−0.6 kb upstream of the translational start site to be important in the activity of a luciferase reporter construct in human dopaminergic SH-SY5Y cells. Moreover, we identified a metal response element (MRE) in this promoter region and showed that it was regulated by metal transcription factor-1 (MTF-1) binding. We also demonstrated that MTF-1 regulates β-Syn expression at both the gene and protein levels.

Human dopaminergic SH-SY5Y cells are commonly used as a model system in the study of Parkinson's disease (PD). SH-SY5Y cells can either be undifferentiated or differentiated and it has been suggested that there are potential limitations in using the former as a model system in for these studies [26]. However, we have previously shown that undifferentiated SH-SY5Y cells express key dopaminergic markers [27], including the dopamine transporter, and were therefore confident in using these cells for the current study.

MTF-1 is a zinc-finger protein transcription factor that induces expression of metallothioneins and other genes involved in metal homeostasis in response to heavy metals such as cadmium, zinc, copper, and silver [28]–[31]. The protein is a nucleocytoplasmic shuttling protein that accumulates in the nucleus upon heavy metal exposure and binds to promoters containing a metal-responsive element (MRE; core consensus sequence TGCRCNC) [32]. MTF-1 is also believed to be activated under other stressful conditions, other than heavy metal load, including oxidative stress and hypoxia [33].

Emerging evidence now suggests that metal homeostasis is strongly linked to the oxidative stress response in many cells types [34]. PD is a progressive neurodegenerative disorder in which oxidative stress and metal toxicity is implicated as a major causative factor [35], [36]. Parkin, a ubiquitin ligase, has been linked to familial form of PD. A *Drosophila* disease model lacking Parkin, given zinc-supplemented food showed improved condition, including extended lifespan and improved motoric abilities [37]. As a zinc-sensing protein, MTF-1 could conceivably be a way through which zinc-supplementation manifests its positive effects. Moreover, overexpression of MTF-1 was found to improve the life-span of Drosophila exposed to oxidative stress and metal toxicity suggesting a common mechanism for protection against both oxidative stress and metal toxicity [38]. Therefore, MTF-1 could potentially have beneficial effect in synucleinopathies.

There is currently no literature suggesting a role of β-Syn in relation to metal homeostasis. However, β-Syn has been suggested to bind copper [39], [40]. A recent study has shown that it has similar affinity for copper as α-Syn and that the co-ordination of copper binding is similar [40]. α-Syn not only binds copper but copper binding increases its aggregation [41]–[43] and results in the formation of toxic oligomeric species that kill cells both exogenously and endogenously [22], [27]. As β-Syn has been shown to decrease the aggregation of α-Syn [44], it is conceivable that this process might involve prevention of copper binding to α-Syn by either binding copper itself or a physical interaction with α-Syn that inhibits copper binding. Alternatively, β-Syn might out-compete α-Syn for copper due to increased expression. In this study we showed that copper appears necessary for β-syn promoter function, as well MTF-1 induction of the β-Syn promoter. Indeed, MTF-1 has been shown to act as a copper-sensing transcriptional activator to regulate human prion gene expression [45], a protein also relevant to neurodegeneration. Increased expression/activity of MTF-1 in response to altered copper levels in the cell could conceivably result in increased levels of β-Syn, protecting the cell from adverse copper binding by α-Syn that could lead to the formation of toxic copper binding oligomers.

MTF-1 altered β-Syn gene regulation and mRNA and protein expression levels. As can be observed in Figure 4 there was a marked increase in protein expression (∼30-fold) when compared to the increase in mRNA expression (∼2-fold). Simply measuring an mRNA expression change does not necessary reflect a change in protein expression [46]. There is support in the literature for discrepancies between mRNA and corresponding protein levels; Pradet-Balade *et al*. (2001) suggested that the abundance of a protein at a given mRNA expression level may vary 30-fold [47]. Furthermore, in Figure 4A the control level (no MTF-1) is already relatively high but this doesn't lead to protein expression that can be detected in the western blot (Figure 4B). This is likely because the turnover of β-Syn mRNA is quite rapid and the protein synthesis machinery is unable to compete, but in the presence of MTF-1 the rate of mRNA synthesis is greater which leads to significant amounts of mRNA being utilised for protein expression. Thus the apparent doubling of mRNA is not informative as to the expected level of protein expression..

As previously highlighted, β-Syn may be a natural negative regulator of the disease-associated α-Syn. Furthermore, transgenic mice over-expressing β-Syn, showed a marked reduction in α-Syn aggregation and protein expression [44]. The importance of the balance between levels of the synucleins is highlighted by the observation that β-Syn inhibits α-Syn aggregation *in vitro* and *in vivo* [2], [48], [49]. It's conceivable that due to cellular stresses, such as heavy metal toxicity or oxidative stress, MTF-1 expression is induced, which consequently alters the regulation of several genes, including β-Syn. β-Syn, in turn, could potentially prevent α-Syn aggregation and/or toxic species. This process may be disrupted in patients with synucleinopathies, as in contrast to control patients, there is a dramatic increase in α-Syn and decrease in β-Syn mRNA levels in the substantia nigra of PD, DLBD and a LB variant of AD patients [14]. Therefore MTF-1 could provide a potential target for disease therapy and this needs to be explored further.

## References

[1] Tabner BJ, Turnbull S, El-Agnaf OM, Allsop D (2002). Formation of hydrogen peroxide and hydroxyl radicals from A(beta) and alpha-synuclein as a possible mechanism of cell death in Alzheimer's disease and Parkinson's disease.. Free Radic Biol Med.

[2] Uversky VN, Li J, Bower K, Fink AL (2002). Synergistic effects of pesticides and metals on the fibrillation of alpha-synuclein: implications for Parkinson's disease.. Neurotoxicology.

[3] Dexter DT, Wells FR, Lees AJ, Agid F, Agid Y (1989). Increased nigral iron content and alterations in other metal ions occurring in brain in Parkinson's disease.. J Neurochem.

[4] Snyder H, Mensah K, Theisler C, Lee J, Matouschek A (2003). Aggregated and monomeric alpha-synuclein bind to the S6' proteasomal protein and inhibit proteasomal function.. J Biol Chem.

[5] McNaught KS, Jenner P (2001). Proteasomal function is impaired in substantia nigra in Parkinson's disease.. Neurosci Lett.

[6] Farrer M, Kachergus J, Forno L, Lincoln S, Wang DS (2004). Comparison of kindreds with parkinsonism and alpha-synuclein genomic multiplications.. Ann Neurol.

[7] West AB, Zimprich A, Lockhart PJ, Farrer M, Singleton A (2001). Refinement of the PARK3 locus on chromosome 2p13 and the analysis of 14 candidate genes.. Eur J Hum Genet.

[8] Nishioka K, Hayashi S, Farrer MJ, Singleton AB, Yoshino H (2006). Clinical heterogeneity of alpha-synuclein gene duplication in Parkinson's disease.. Ann Neurol.

[9] Jakes R, Spillantini MG, Goedert M (1994). Identification of two distinct synucleins from human brain.. FEBS Lett.

[10] Biere AL, Wood SJ, Wypych J, Steavenson S, Jiang Y (2000). Parkinson's disease-associated alpha-synuclein is more fibrillogenic than beta- and gamma-synuclein and cannot cross-seed its homologs.. J Biol Chem.

[11] Ohtake H, Limprasert P, Fan Y, Onodera O, Kakita A (2004). Beta-synuclein gene alterations in dementia with Lewy bodies.. Neurology.

[12] Galvin JE, Uryu K, Lee VM, Trojanowski JQ (1999). Axon pathology in Parkinson's disease and Lewy body dementia hippocampus contains alpha-, beta-, and gamma-synuclein.. Proc Natl Acad Sci U S A.

[13] Li W, Lesuisse C, Xu Y, Troncoso JC, Price DL (2004). Stabilization of alpha-synuclein protein with aging and familial parkinson's disease-linked A53T mutation.. J Neurosci.

[14] Rockenstein E, Hansen LA, Mallory M, Trojanowski JQ, Galasko D (2001). Altered expression of the synuclein family mRNA in Lewy body and Alzheimer's disease.. Brain Res.

[15] Mori F, Nishie M, Yoshimoto M, Takahashi H, Wakabayashi K (2003). Reciprocal accumulation of beta-synuclein in alpha-synuclein lesions in multiple system atrophy.. Neuroreport.

[16] Shen Q, Wang Y, Dimos JT, Fasano CA, Phoenix TN (2006). The timing of cortical neurogenesis is encoded within lineages of individual progenitor cells.. Nat Neurosci.

[17] Kruger R, Vieira-Saecker AM, Kuhn W, Berg D, Muller T (1999). Increased susceptibility to sporadic Parkinson's disease by a certain combined alpha-synuclein/apolipoprotein E genotype.. Ann Neurol.

[18] Xia Z, DePierre JW, Nassberger L (1996). Dysregulation of bcl-2, c-myc, and Fas expression during tricyclic antidepressant-induced apoptosis in human peripheral lymphocytes.. J Biochem Toxicol.

[19] Chiba-Falek O, Nussbaum RL (2001). Effect of allelic variation at the NACP-Rep1 repeat upstream of the alpha-synuclein gene (SNCA) on transcription in a cell culture luciferase reporter system.. Hum Mol Genet.

[20] McHugh PC, Rogers GR, Loudon B, Glubb DM, Joyce PR (2008). Proteomic analysis of embryonic stem cell-derived neural cells exposed to the antidepressant paroxetine.. J Neurosci Res.

[21] Brown DR (2004). Role of the prion protein in copper turnover in astrocytes.. Neurobiol Dis.

[22] Wang X, Moualla D, Wright JA, Brown DR (2010). Copper binding regulates intracellular alpha-synuclein localisation, aggregation and toxicity.. J Neurochem.

[23] Lavedan C, Leroy E, Torres R, Dehejia A, Dutra A (1998). Genomic organization and expression of the human beta-synuclein gene (SNCB).. Genomics.

[24] Schug J (2008). Using TESS to predict transcription factor binding sites in DNA sequence.. Curr Protoc Bioinformatics. Vol. Chapter 2.

[25] Roth S, Zhang S, Chiu J, Wirth EK, Schweizer U (2010). Development of a serum-free supplement for primary neuron culture reveals the interplay of selenium and vitamin E in neuronal survival.. J Trace Elem Med Biol.

[26] Xie HR, Hu LS, Li GY (2010). SH-SY5Y human neuroblastoma cell line: in vitro cell model of dopaminergic neurons in Parkinson's disease.. Chin Med J (Engl).

[27] Wright JA, Wang X, Brown DR (2009). Unique copper-induced oligomers mediate alpha-synuclein toxicity.. Faseb J.

[28] LaRochelle O, Labbe S, Harrisson JF, Simard C, Tremblay V (2008). Nuclear factor-1 and metal transcription factor-1 synergistically activate the mouse metallothionein-1 gene in response to metal ions.. J Biol Chem.

[29] Heuchel R, Radtke F, Georgiev O, Stark G, Aguet M (1994). The transcription factor MTF-1 is essential for basal and heavy metal-induced metallothionein gene expression.. Embo J.

[30] Serfling E, Lubbe A, Dorsch-Hasler K, Schaffner W (1985). Metal-dependent SV40 viruses containing inducible enhancers from the upstream region of metallothionein genes.. Embo J.

[31] Stuart GW, Searle PF, Palmiter RD (1985). Identification of multiple metal regulatory elements in mouse metallothionein-I promoter by assaying synthetic sequences.. Nature.

[32] Saydam N, Georgiev O, Nakano MY, Greber UF, Schaffner W (2001). Nucleo-cytoplasmic trafficking of metal-regulatory transcription factor 1 is regulated by diverse stress signals.. J Biol Chem.

[33] Lichtlen P, Schaffner W (2001). The "metal transcription factor" MTF-1: biological facts and medical implications.. Swiss Med Wkly.

[34] Shinyashiki M, Chiang KT, Switzer CH, Gralla EB, Valentine JS (2000). The interaction of nitric oxide (NO) with the yeast transcription factor Ace1: A model system for NO-protein thiol interactions with implications to metal metabolism.. Proc Natl Acad Sci U S A.

[35] Uttara B, Singh AV, Zamboni P, Mahajan RT (2009). Oxidative stress and neurodegenerative diseases: a review of upstream and downstream antioxidant therapeutic options.. Curr Neuropharmacol.

[36] Bolognin S, Messori L, Zatta P (2009). Metal ion physiopathology in neurodegenerative disorders.. Neuromolecular Med.

[37] Saini N, Schaffner W (2010). Zinc supplement greatly improves the condition of parkin mutant Drosophila.. Biol Chem.

[38] Bahadorani S, Mukai S, Egli D, Hilliker AJ (2010). Overexpression of metal-responsive transcription factor (MTF-1) in Drosophila melanogaster ameliorates life-span reductions associated with oxidative stress and metal toxicity.. Neurobiol Aging.

[39] Binolfi A, Lamberto GR, Duran R, Quintanar L, Bertoncini CW (2008). Site-specific interactions of Cu(II) with alpha and beta-synuclein: bridging the molecular gap between metal binding and aggregation.. J Am Chem Soc.

[40] Davies P, Wang X, Sarell CJ, Drewett A, Marken F (2010). The Synucleins are a Family of Redox Active Copper Binding Proteins.. Biochemistry.

[41] Uversky VN, Li J, Fink AL (2001). Metal-triggered structural transformations, aggregation, and fibrillation of human alpha-synuclein. A possible molecular NK between Parkinson's disease and heavy metal exposure.. J Biol Chem.

[42] Paik SR, Shin HJ, Lee JH, Chang CS, Kim J (1999). Copper(II)-induced self-oligomerization of alpha-synuclein.. Biochem J.

[43] Lee JC, Gray HB, Winkler JR (2008). Copper(II) binding to alpha-synuclein, the Parkinson's protein.. J Am Chem Soc.

[44] Fan Y, Limprasert P, Murray IV, Smith AC, Lee VM (2006). Beta-synuclein modulates alpha-synuclein neurotoxicity by reducing alpha-synuclein protein expression.. Hum Mol Genet.

[45] Bellingham SA, Coleman LA, Masters CL, Camakaris J, Hill AF (2009). Regulation of prion gene expression by transcription factors SP1 and metal transcription factor-1.. J Biol Chem.

[46] Anderson L, Seilhamer J (1997). A comparison of selected mRNA and protein abundances in human liver.. Electrophoresis.

[47] Pradet-Balade B, Boulme F, Mullner EW, Garcia-Sanz JA (2001). Reliability of mRNA profiling: verification for samples with different complexities.. Biotechniques.

[48] Hashimoto M, Rockenstein E, Mante M, Mallory M, Masliah E (2001). beta-Synuclein inhibits alpha-synuclein aggregation: a possible role as an anti-parkinsonian factor.. Neuron.

[49] Park JY, Lansbury PT (2003). Beta-synuclein inhibits formation of alpha-synuclein protofibrils: a possible therapeutic strategy against Parkinson's disease.. Biochemistry.

[50] Wright JA, McHugh PC, Stockbridge M, Lane S, Kralovicova S (2009). Activation and repression of prion protein expression by key regions of intron 1.. Cell Mol Life Sci.

[51] Wang X, Moualla D, Wright JA, Brown DR (2010). Copper binding regulates intracellular alpha-synuclein localisation, aggregation and toxicity.. J Neurochem.

